# Beyond the coach: contextual, community, and organizational predictors of coaches’ perceived impact on athlete life skill development

**DOI:** 10.3389/fspor.2026.1901614

**Published:** 2026-08-26

**Authors:** Samantha Bates, Dawn Anderson-Butcher, Kylee J. Ault-Baker, Emily A. Nothnagle

**Affiliations:** College of Social Work, The Ohio State University, Columbus, OH, United States

**Keywords:** coaching, equity, life skill development, organizational support, positive youth development, youth sport

## Abstract

**Introduction:**

Youth sport is widely regarded as a context for promoting positive youth development (PYD) and life skill development; however, inequities in the youth sport system can limit coaches' perceived ability to foster these outcomes. Guided by an integrated youth sport system perspective, this study examined how contextual factors, community-level resource access, and organizational training and support were associated with coaches' perceived impact on athlete life skill development.

**Methods:**

Using data from the U.S. National Coach Survey (*N* = 4,964), hierarchical linear regression analyses were conducted with predictors entered in three blocks: (1) context (school vs. community setting, urbanicity, proportion of athletes experiencing poverty, proportion of athletes identifying as Black, Latino, Asian/Pacific Islander, or multiracial), (2) access to community-level resources (e.g., equipment, transportation, safe practice spaces), and (3) organizational support and training [coaching certification, life skills training, administrative support, peer support, compensation (paid vs. unpaid), and having been evaluated last season].

**Results:**

Context accounted for 5.4% of the variance in perceived impact. Access to community-level resources accounted for an additional 1.7% of the variance, with equipment availability emerging as a significant predictor. Organizational support and training variables accounted for the largest increase in variance (Δ*R*^2^ = 6.5%, *p* < .01), with training in life skill development through sport, peer support, coaching certification, paid status, and prior evaluation associated with greater perceived impact on life skill development (*R*^2^ = .136).

**Discussion:**

Findings suggest that coaches' capacity to support life skill development can be shaped by factors within youth sport organizations and the broader youth sport context. More research is needed to understand drivers of coaching to support life skill development. Implications for practice, policy, and coach education are discussed.

## Introduction

With more than 60 million youth participating in sport annually in the United States (U.S.), including approximately 8 million high school athletes (1, 2), sport has the potential to promote mental, physical, and social well-being at a broad scale. Indeed, sport is widely recognized as a context for fostering positive youth development [PYD (1, 3–5)]. However, participation alone does not always result in positive developmental outcomes (6, 7). Instead, the developmental benefits of sport are largely the result of the quality of relationships, sport program design, and broader contextual conditions of youth sport experiences (8–12). While the importance of broader factors influencing youth outcomes is increasingly recognized, gaps remain in how contextual, community, and organizational inequities and protective factors affect a coach's ability to leverage sport as a context for PYD.

One of the most reported PYD outcomes related to sport involvement and coaching is life skill development. Life skills are defined as “interpersonal assets, characteristics, and skills that can be facilitated or developed in sport and transferred to non-sport settings” (10, p. 60). Life skills develop in a variety of sport settings such as community sport, school sport programs, and sport-based PYD (SBPYD) programs. For instance, Bruner et al. (13) conducted a meta-analysis of SBPYD interventions and found that overall, there were significant improvements in life skill development among youth participants. Studies in school and community sport settings have identified gains in communication, emotional regulation, leadership, time management, and work ethic (14–18). Ultimately, researchers stress life skill development does not just happen while participating in sport. Rather, it is influenced by the context and agents within that context.

Contextual factors such as caring and mastery-oriented climates have been linked to positive life skill development and overall positive PYD outcomes, whereas ego-oriented climates have been associated with less positive developmental experiences (19–21). Coaches, parents/caregivers, teammates, and other positive adults are also widely recognized as important mechanisms for modeling, reinforcing, and transferring life skills beyond the sport context (14, 22, 23). Moreover, intentional coaching practices, reinforcement techniques, and structured curricula are shown to be effective in enhancing life skill development in sport programs (8, 23–25).

As evidenced by the examples above, coaches are key agents who play a significant role in both positively and negatively supporting the development of life skills through sport (22, 26–28). Coaching practices that encourage autonomy-supportive, caring, mastery-oriented coaching will be more likely to enhance life skills and psychological well-being (28, 30), while controlling and unsupportive coaching practices will be more likely to undermine it (14, 31). Equally important to the climate is the extent to which coaches intentionally support the transfer of life skills beyond the playing field, court, mat, or pitch, commonly referred to as life skill transfer (28). When coaches facilitate the transfer of life skills, athletes are more likely to apply skills across domains, including school, home, peer relationships, and future employment settings (28, 32, 33).

While studies are promising for advancing PYD through sport, coaches’ implementation of practices supportive of life skill development does not occur in isolation. Coaching practices and behaviors are influenced by wider and interdependent contextual, community, and organizational factors within which coaches work (4, 28). Few studies have used large datasets to identify factors related to coaches’ perceptions of their impact on athlete life skill development (22). This gap is particularly important given growing calls to position sport as a public health strategy to promote physical, mental, social, and emotional well-being among young people. If sport is to fulfill this potential, researchers and practitioners must move beyond the coach to examine the broader systems that support or constrain life skill development through sport.

## Contextual, community, and organizational influences within the youth sport system

Youth sport is increasingly recognized as a complex developmental system in which outcomes are shaped through interactions among athletes, coaches, families, organizations, communities, and broader policy environments (4). Although coaches are often identified as primary mechanisms through which positive youth development (PYD) occurs, their ability to facilitate life skill development does not occur in isolation. Rather, coaching practices are embedded within organizational, community, and societal contexts that provide varying levels of resources, support, training, and opportunity (34). Consequently, sport settings differ in their capacity to foster PYD, suggesting that coaches’ perceived impact on life skill development may be influenced not only by individual knowledge and behaviors but also by the broader systems in which they operate.

Access to high-quality sport experiences and well-prepared coaches may vary by urbanicity (e.g., suburban, rural, urban) and other contextual factors (34). For example, youth living in urban or rural communities who experience the effects of poverty and/or historical marginalization often participate in organized sports at lower rates, largely due to structural barriers related to affordability, transportation, and access rather than differences in interest (2, 35). Consequently, where youth live and where they engage in sport may dictate the quality of their experience and the extent to which the environment is intentionally designed to foster PYD.

Governance structures and policies also influence the youth sports system, which shapes whether and to what extent coaches are supported and prepared to facilitate PYD. In school-based sport settings, coaches operate within more formalized systems of oversight, including state-mandated training requirements and interscholastic governing bodies such as the National Federation of State High School Associations, which emphasize education-based athletics and often explicitly identify life and leadership skill development as core values (36, 37). Because of these oversight systems, many school-based coaches in the U.S. are required to complete standardized training (e.g., CPR, First Aid, concussion protocols), with an increasing number of states also requiring education in pedagogy, PYD, and mental health (38, 39). These systems frequently include accountability mechanisms and credentialing pathways, such as licensure, continuing education requirements, or compliance monitoring, which create more consistent expectations for coach preparation (36, 38).

In contrast, community-based sport (e.g., recreational, developmental, competitive, club) often operates within a more decentralized and loosely regulated structure. Policies guiding coach preparation may be limited, and in many cases, coaching requires minimal formal training or credentialing (36, 38). As a result, substantial variation may exist in coaches’ knowledge, skills, and access to professional development opportunities focused on PYD and life skill development (34). Emerging evidence on the efficacy of school-based coaches compared with community-based coaches supports this distinction, with school-based coaches reporting higher levels of preparedness to support PYD than community-based coaches (40).

A community's socioeconomic and demographic makeup may further shape the potential of sport to facilitate PYD via access to resources. Highly trained or experienced coaches may be disproportionately concentrated in more well-resourced communities where compensation, organizational infrastructure, and professional development opportunities are more readily available (2, 41). Alternatively, sport programs in under-resourced communities, particularly those serving higher proportions of youth experiencing economic hardship or historically marginalized youth, may face structural barriers related to facilities, equipment, staffing, transportation, and access to high-quality training opportunities (34). In these settings, immediate demands, such as maintaining participation, ensuring safety, securing facilities, or acquiring equipment, may take precedence over investments in coach development and facilitation of PYD practices. At the same time, sport may serve an important developmental role in under-resourced communities by providing youth with opportunities to build relationships, develop life skills, and access supportive environments that extend beyond school and home contexts (8, 11, 32).

Organizational constraints or opportunities may further limit or support coaches’ ability to deliver high-quality sport experiences and promote life skill development. For example, scholars found that coaches across compensation levels (i.e., volunteer, part-time, and full-time) experience organizational stressors such as limited funding, inadequate facilities and equipment, and administrative burden (42). These constraints increased strain on coaches but may also reduce their capacity to intentionally teach and reinforce life skills through sport. Conversely, organizational supports, including access to educational initiatives, administrative support, and financial resources, have been shown to increase coaches’ motivation to engage in professional development (43). Coach training requirements, certification pathways, administrative support, access to facilities and equipment, transportation, and investments in professional development may all influence coaches’ capacity to deliver high-quality sport experiences and intentionally promote PYD outcomes (4, 36, 41, 44). Examples suggest organizational resources can foster coach learning and behaviors conducive to promoting PYD outcomes.

Lastly, coach education is often cited as a key strategy for enhancing coaches’ ability to promote life skill development. Research suggests coach education on life skill development through sport can improve both coaching practices and athlete outcomes (22, 27, 28, 30, 45–47). However, access to such training opportunities remains uneven (34). Barriers related to cost, availability, time, and organizational capacity may limit participation, particularly within under-resourced communities and sport organizations (41). According to the U.S. National Coach Survey, 66% of coaches report past participation in training focused specifically on life skill development through sport (34). Despite understanding the prevalence of those trained, less is known about whether coach education alone is a protective factor in the absence of broader contextual, community, and organizational inequities or assets within the youth sport system.

## Current study

Much of the existing literature on coaches’ perceptions of life skill development has examined coaching efficacy or training participation, with comparatively limited attention to the multi-level conditions that shape those behaviors and their perceived impact on athletes may emerge (22, 27, 34, 48). As a result, gaps exist in understanding how broader factors influence coaches’ perceptions and abilities that, in turn, can be used as leverage points to promote equitable access to high-quality, developmentally enriching sport experiences for more youth. Additionally, there remains a lack of large-scale national research capturing systems-level factors on the conditions that facilitate life skill development from the coach perspective (22). Addressing these gaps can help identify leverage points to strengthen youth sport systems, improve coaching effectiveness, and expand equity and access to PYD-oriented sports environments for more young people, including those most vulnerable.

## Methods

### Procedures

Participants were recruited through the U.S. National Coach Survey, which builds on prior LiFE*sports* research measuring coaching practices in Central Ohio and statewide contexts (8, 40, 49). Recruitment materials, including a standardized script, were disseminated via social media and targeted email outreach to coaches, school personnel, athletic administrators, sports organizations, coach education platforms, and other stakeholders across both the nonprofit and commercial sport sectors. Data were collected online using Qualtrics between March and October 2022. Given the focus of the National Coach Survey was to reach thousands of coaches and measure multiple constructs of interest, single item measures were used for many variables to keep the survey shorter and maintain face validity, thereby reducing repetitiveness and increasing the likelihood of completion [see (50, 51)]. Prior to accessing the survey, participants completed an electronic informed consent process. As an incentive, one respondent was randomly selected to receive a $50 electronic gift card for every 500 surveys in which an email address was provided. All study procedures were reviewed and approved by The Ohio State University Institutional Review Board.

### Sample

The final analytic sample (*N* = 4,964) included coaches who provided complete data on the outcome variable and valid responses on at least one predictor. Most respondents were affiliated with school-based sport programs, with approximately 48% coaching in school settings and 83% serving in head coach roles. The sample was predominantly male (76%), with a mean age of 45.71 years (SD = 11.22). In terms of racial and ethnic identity, 80% identified as White, 4% as African American, 7% as Hispanic/Latino, 2% as Asian American, 3% as multiracial, and 1% as Native American or American Indian; the remaining participants did not report their race (See Table 1).

**Table 1:** Descriptive statistics (*N* = 4,964).

Variable	%	Mean (SD)	Range
Age (Years)		45.71 (11.22)	18–81
Gender			
Male	75.6		
Female	24.4		
Race/Ethnicity			
White	80.2		
Hispanic/Latino	6.6		
Black/African American	4.2		
Asian/Pacific Islander	2.0		
Multiple races	3.2		
Other/prefer not to answer	2.8		
Primary Coaching Setting			
School-based	47.6		
Community-based	52.4		
Geographic Context			
Rural/Town	50.3		
Urban	19.8		
Suburban	29.9		
% Team identified as youth of color		23.79 (31.39)	0–100
% Team experiencing economic hardship		20.79 (26.88)	0–100
Equipment		76.95 (22.95)	0–100
Transportation		76.84 (27.30)	0–100
Safe space to practice		83.84 (23.25)	0–100
Administrative support		70.59 (29.12)	0–100
Peer support		72.69 (26.11)	0–100
Certification			
Certified	56.4		
Not certified	43.6		
Training in Life Skill Development			
Prior participation	60.2		
No prior participation	39.8		
Evaluated last season			
Yes	54.1		
No	45.9		
Paid Status			
Paid	63.5		
Unpaid	36.5		
Impact on athletes developing life skills		74.77 (19.95)	0–100

SD,  standard deviation.

### Measures

#### Perceived impact on athletes’ life skill development

The dependent variable captured coaches’ perceived impact on athletes’ life skill development. Coaches responded to the item: “In the setting where you MOST OFTEN coach your PRIMARY sport, how much of an impact have you had on your athletes: Developing life skills?” Responses were provided on a sliding scale ranging from 0 to 100, with higher scores indicating greater perceived impact.

#### Context

Several variables were used to represent the context in which coaches operated. Sport setting was measured as whether coaches primarily coached in a school-based (coded 1) or a community-based setting (coded 0; reference group). Geographic context was represented by two dummy variables: rural/town (coded as 1) and urban settings (coded as 1), with suburban (coded as 0) serving as the reference group. Coaches reported the percentage of athletes on their teams experiencing economic hardship (0%–100%) and the percentage identified as youth of color (e.g., Black, Latino; Multi-racial 0%–100%). Both variables were treated as continuous indicators in the model, with higher values reflecting greater concentrations of economic disadvantage and racial/ethnic diversity among youth living in the communities where sport was taking place. Importantly, these measures were conceptualized as proxies of the broader community context in which coaching occurs. Rather than representing individual-level characteristics, they capture variation in the social and structural conditions shaping athletes’ experiences, including access to resources, exposure to inequities, and community-level demographic characteristics. As such, these variables allow for examination of how coaching processes and outcomes may differ across contexts characterized by varying levels of economic hardship and racial/ethnic diversity.

#### Community-level resources

Access to community resources was measured using three items assessing the extent to which coaches had adequate equipment, transportation, and safe practice spaces during the previous season. Each item asked coaches, “Move the bar to the right or left to indicate to what degree did you have enough of the following resources during your LAST SEASON when coaching your PRIMARY sport..” was rated on a 0–100 scale, with higher scores indicating greater perceived access.

#### Organizational support and training

Organizational factors included perceived administrative support and peer support measured by an item reading, “Move the bar to the right or left to indicate to what degree you had enough of the following resources during your LAST SEASON when coaching your PRIMARY sport..” The question then asked, “Administrative support” and “Peer support,” and participants rated their perceptions of support on a scale of 0–100, with higher scores indicating greater perceived support. Coaches reported whether they held a formal coaching certification, with certification coded as 1 and not being certified as 0 (e.g., reference group). Another item assessed whether coaches had participated in training focused on life skill development through sport (e.g., prior participation coded as 1 and no prior participation coded as 0). Finally, coaches were asked whether they had been formally evaluated during the previous season (e.g., being evaluated last season was coded as 1, and not being evaluated last season was coded as 0). Paid status was measured dichotomously to indicate whether the coach was paid (coded as 1) or unpaid (coded as 0) for their coaching role last season when coaching their PRIMARY sport.

#### Analytic strategy

Prior to primary analyses, data were screened for missingness, normality, and multicollinearity. Descriptive statistics and bivariate correlations were examined for all study variables to assess distributions and relationships among predictors and the outcome variable. Missing data were handled using listwise deletion, retaining only cases with complete data across the variables included in the regression models. Listwise deletion was selected to maintain consistency across hierarchical regression models to examine factors associated with coaches’ perceived impact on athlete life skill development. All linear regression assumptions were evaluated and met, including variance inflation factors, which were assessed as each block was added to the model. Collinearity diagnostics indicated no concerns (all VIFs < 1.60). Variables were entered in three blocks: (1) contextual, (2) community resource access, and (3) organizational support and training. This stepwise approach allowed for examination of the incremental variance explained by contextual, community, and organizational factors. Standardized regression coefficients (*β*) were used to assess the relative strength of predictors, and statistical significance was set at *p* < .05. Prior to analysis, data were screened for missingness, normality, multicollinearity, and outliers.

## Results

In the first block, context variables (setting, urbanicity, proportion of athletes experiencing poverty, and proportion of athletes who identify as racially/ethnically diverse) were entered. The model was significant, *F*(5, 4958) = 56.66, *p* < .001, and accounted for 5.4% of the variance in perceived impact on life skill development (*R^2^* = .054, adjusted *R^2^* = .053). Coaches working in school-based settings reported significantly higher perceived impact than those in community-based settings (*β* = .182, *p* < .001). Coaches with teams with a greater proportion of athletes experiencing poverty (*β* = .065, *p* < .001) and greater racial/ethnic diversity (*β* = .047, *p* = .002) reported higher perceived impact. Urban location was associated with slightly lower perceived impact compared to suburban settings (*β* = −.036, *p* = .025), whereas rural/town location was non-significant (*p* = .230). Overall, the effect size for this block of variables was small (*f^2^* = .057)

In the second block, access to transportation, safe practice spaces, and equipment were added. This model was also significant, *F*(8, 4955) = 47.58, *p* < .001, and accounted for an additional 1.7% of the variance (*ΔR^2^* = .017, *p* < .001; total *R^2^* = .071), representing a very small incremental effect (*f^2^* = .018). Greater access to equipment was a significant positive predictor (*β* = .102, *p* < .001). Transportation (*β* = .030, *p* = .046) and safe practice spaces (*β* = .032, *p* = .034) were also statistically significant, although the effects were small. Structural context effects remained largely consistent, with school-based settings and higher poverty levels among athletes continuing to predict greater perceived impact.

The final model added organizational support and training variables (certification, life skills training, administrative support, peer support, paid status, and evaluation). This model was significant, *F*(14, 4949) = 55.45, *p* < .001, and accounted for the largest increase in variance (*ΔR^2^* = .064, *p* < .001), with a total adjusted *R^2^* of.133, representing a small to medium overall effect across all three blocks [*f^2^* = .157 (6)]. Several organizational factors were significant predictors of greater perceived impact of life skill development: Life skills training (*β* = .149, *p* < .001); Paid coaching status (*β* = .144, *p* < .001); Peer support (*β* = .112, *p* < .001); Being evaluated in the prior season (*β* = .044, *p* = .002); and Coaching certification (*β* = .027, *p* = .045) In this model, access to equipment remained significant (*β* = .076, *p* < .001), though its effect was reduced. Transportation and safe practice space were no longer significant. Among contextual variables, school-based settings (*β* = .068, *p* < .001), higher team compositions with athletes experiencing economic hardship (*β* = .066, *p* < .001), and greater racial/ethnic diversity (*β* = .028, *p* = .045) remained positively associated with perceived impact, while urban location remained a small negative predictor (*β* = −.036, *p* = .019). Rural/town location and administrative support were non-significant in the final model. Results from each model are reported in Table 2.

**Table 2:** Hierarchical linear regression predicting coaches’ perceived impact on athlete life skill development (*N* = 4,964).

Predictor	Model 1 *β*	Model 2 *β*	Model 3 *β*
Block 1: Context			
School-based setting (vs. community)	.182**	.173**	.068**
Rural/town (vs. suburban)	.020	.020	.023
Urban (vs. suburban)	−.036*	−.029	−.036*
% Athletes experiencing economic hardship	.065**	.084**	.066**
% Athletes who identify as youth of color	.047**	.044**	.028*
Block 2: Community Resources			
Equipment access		.102**	.076**
Transportation access		.030*	.016
Safe practice space		.032*	.005
Block 3: Organizational Support & Training			
Administrative support			−.012
Peer support			.112**
Coaching certification (vs. not certified)			.027*
Life skills training			.149**
Evaluated last season (vs. not evaluated)			.044**
Paid (vs. unpaid)			.144**
**Model *R*^2^**	.054	.071	.136
***ΔR* ^2^**	—	.017**	.064**
**Adjusted *R*^2^**	.053	.070	.133
**Cohen's f^2^**	.057	0.76	.157
***F***	56.66**	47.58**	55.45**

β = standardized coefficient. Suburban and community settings are reference groups. *p < .05. ** *p* < .01.

## Discussion

This study examined contextual, community-level resources, and organizational supports as predictors of coaches’ perceived impact on athlete life skill development. Using data from the U.S. National Coach Survey (34), this study leveraged a large sample to better understand how coaches perceive their influence on athlete life skill development, relative to broader systems shaping the youth sport landscape. Findings from all three models suggest some factors in the environment which may shape coaches’ perceived capacity to facilitate life skill development.

Consistent with prior research, coaches in school-based settings reported significantly greater perceived impact than those in community-based settings (34, 40). One potential explanation for this finding relates to the purpose and structure of school-based sport. School sport has increasingly been framed as an extension of the educational mission, emphasizing student development, leadership, and life skill acquisition alongside athletic performance (37, 52). Guided by school missions and educational priorities, school-based coaches may place greater intentional emphasis on life skill development and broader youth outcomes. Furthermore, school-based sport settings may provide both contextual and organizational supports that facilitate coaches’ engagement in PYD practices (40). For example, school-based coaches across all 50 states are required to participate in some form of coach education and training through state laws and interscholastic governing bodies (30, 36–38, 53).

Given that both coach certification and training were related to greater perceived impact on athlete life skill development, findings may suggest that policy and training infrastructures embedded within school sport systems help support coaching practices aligned with PYD outcomes. At the same time, prior research indicates that only a limited proportion of required coach education content focuses specifically on PYD or social-emotional learning (36), and as of 2025, only two states required standalone PYD-related coach education (38). Thus, while existing systems of oversight and training may contribute to stronger developmental climates in school-based settings, if policies are protective, findings point to opportunities to strengthen PYD-related coach education more broadly.

In contrast, community-based sport settings are often more decentralized and less regulated, with fewer formalized coach training requirements and accountability structures (40). As such, coaches in these settings may experience greater variability in preparation, organizational support, and access to professional development opportunities related to developing life skills among their athletes. Findings point to potential opportunities to strengthen community-based coaches’ values toward and practices focused on life skill development, which may require further alignment of policies that go beyond sport tactics, techniques, and instruction and toward a more holistic perspective, inclusive of PYD-related training requirements (38, 40). If policies requiring training and governance structures enable coaches to make a broader impact on athletes, perhaps even younger athletes, then sport may do more to fulfill its potential as a platform for PYD and prevention.

Our results also indicate that coaches working in contexts with higher proportions of athletes experiencing poverty and greater racial/ethnic diversity reported significantly greater perceived impact on athlete life skill development. Sport when designed to build life skills and address broader social problems is especially important for youth experiencing social and structural vulnerabilities (3, 8, 22, 24, 54). Findings may point to how coaches in highly impacted communities may be more likely to emphasize life skill development and broader PYD outcomes and also perhaps coaching in contexts where youth may be limited in these skillsets and there is the greatest need for building these assets. Further research should examine how community demographics shape more tangible coaching behaviors, priorities, and developmental outcomes to better understand the mechanisms underlying these relationships.

Access to equipment, transportation, and safe practice spaces emerged as significant positive predictors of coaches’ perceived impact on athlete life skill development in the second model. Perhaps when coaches are not burdened by securing the basic resources necessary for participation, they may perceive themselves to have greater capacity to focus on relationship-building, intentional instruction, and other developmental aspects of coaching. In this way, equity and access to basic needs in facilitating sport may underpin whether sport can be fully maximized to promote PYD. However, findings from the third model, which included organizational support and coach training as predictors, suggest that developmental infrastructures within sport organizations may be the most influential in shaping coaches’ perceived influence on athletes’ life skill development. These findings align with prior literature in which organizational supports, professional development, and workforce investments were shown to strengthen coaches’ motivation, preparedness, and implementation of PYD-related practices (41, 43, 47).

Here, the coaches who reported access to certification and training opportunities, particularly those explicitly focused on life skill development, also reported significantly greater perceived impact. Others have amplified this finding, suggesting certification and coach education may provide coaches with strategies, language, and frameworks for integrating life skill instruction into sport settings while also reinforcing broader organizational expectations regarding PYD (22, 27, 30, 47, 50, 55–57). In this way, coach education may function not only as a mechanism for skill acquisition but also as an organizational signal that teaching life skills is a valued component of the coaching role.

Additionally, coaches in this study reporting greater peer support, pay, and formal evaluation also reported significantly greater perceived impact on athlete life skill development. These findings align with prior research suggesting that supportive coaching cultures and collaborative learning environments may reinforce PYD-oriented coaching practices through opportunities for mentorship, modeling, and shared learning among coaches (44, 58, 59). Others have found similar results, suggesting that pay, evaluation, and feedback processes promote reflection and adaptation in coaching behaviors leading to improved coaching effectiveness and resultant athlete outcomes (29, 60).

In the end, the exploration of organizational and contextual factors that influence coaching behaviors is an underdeveloped area of coaching research (22, 55). Although this study contributes to this knowledge gap, the final model still only predicts a small amount of variance in coaches’ perceived impact on athletes’ life skill development. There is still much to distill to better understand factors influencing whether coaches focus on life skill development in their coaching behaviors, as well as if they indeed impact athletes’ broader PYD. As examples, organizational climate and culture, as well as sport-specific contextual climate, have been found to be important predictors of social and life skill development among athletes (61, 62). Further, the value coaches place on coaching for life skill development is associated with greater perceived impact on athletes’ life skill development (22). Exploring these and other factors, such as organizational processes, expectations for coaches, and broader social support systems conducive to wholistic coaching, is ripe for further inquiry.

Additionally, sport is organized differently based on type of sport, age, background and skill level of the athletes coached, competitiveness of the context, and other factors. As examples, PYD-related coaching practices are less common at more competitive, advanced sporting contexts (34, 63). Coaching practices to support broader PYD outcomes are more evident when working with youth experiencing social vulnerabilities (3). Distilling how these and other contextual and organizational factors influence coaching philosophies and practices, especially in relation to a focus on life skill development and outcomes, remains a growing priority. In addition, qualities and characteristics of the coach also may be important predict variables, ones such as past lived experiences, previous coaching experiences, and perhaps relational factors evident in their own childhood sport experiences. Study findings do, however, point to some practical implications to guide how sport is organized and structured.

## Implications

This current study illustrates potential ways in which sport organizations, athletic departments, and governing bodies may provide opportunities for coaches to learn, prepare, and increase their capacities to engage in sport experiences that are further PYD-oriented. Study findings also have importance in relation to both education and training. More specifically, coach education was identified as one of the strongest predictors for coaches’ perceived influence in athlete life skill development, indicating that coach education may be a practical means for addressing inequities in sport development experiences in various contexts. There are existing programs, such as *Coach Beyond* (30, 56) and *Coaching for Life Skills* (27, 57), that offer models that have proven promising for scaling online and in-person training to increase coaches’ competencies in PYD and psychosocial development. Making these programs more widely available could equip coaches to actively support the development of life skills and overall athlete development in any context.

Important policy and organizational implications for youth sport systems also may be called for based on study findings. Currently, there is a limited focus on PYD-related competency areas in many coach education requirements, with greater attention paid to physical health and safety topics (e.g., CPR, concussion management) and sport-specific tactics and techniques (36, 38). Expanding requirements for certification and professional development in areas beyond the X's and O's may be a growing priority for athletic departments, governing bodies, and policymakers. Implementing broader national standards for coach education could also facilitate greater uniformity in expectations for coaches in their roles supporting athlete development beyond sport performance and safety. Other options include providing technical assistance to youth sport organizations to align training requirements with broader mission statements and values focused on holistic athlete development. In doing so, access to affordable, low-cost trainings that don't become barriers to on-boarding and retaining coaches will remain critical as improvements in policy and practice are considered.

Meanwhile, access to transportation, equipment, and practice spaces also were related to perceived impact, highlighting the need to eliminate systemic barriers to participation in addition to coach training programs. Findings also call out the importance of perceived life skill impact when coaching youth from socially vulnerable experiences. Collaboration between community agencies and sport organizations can help to mitigate resource disparities that limit coaches’ ability to support athlete development. For instance, *Leveling the Playing Field*, an organization, distributes equipment to under-resourced schools and youth sport organizations to lessen financial barriers to participation. LiFE*sports* at The Ohio State University is offered at no cost to participants, while also providing two meals/day and transportation to most programs. These and other activities could enable coaches to focus more on PYD-oriented coaching than on identifying basic resources and addressing access issues. However, if there are no alternatives to pay-to-play sports systems, inequities in access to high-quality sports experiences are likely to persist.

Lastly, relationships also were found among peer support, evaluations, compensation, and perceived impact on life skill development, pointing to the need to move beyond one-off coach training and invest in broader developmental pathways for coaches that facilitate continuous coach development and retention. Facilitating peer mentoring, collaborative learning groups, formal feedback, and reflection on practice could support coaches to implement PYD-oriented strategies over time. In the same way, reward systems that incentivize coaches to take time to help athletes develop beyond their sport performance (e.g., acting as leaders in the community, doing well academically, demonstrating respect/sportsmanship) may further support the translation of life skill development as an expected outcome of the sport experience. Overall, our results add to the existing research and policy discussion on maximizing sport as a context for PYD, highlighting ways in which sport organizations, communities, and governing systems can design and support coaches to promote athletes’ life skill development.

## Limitations

Despite the contributions of this study, several limitations remain which should be considered when interpreting the study findings. All participants volunteered to participate in the U.S. National Coach Survey and were not randomly selected nor geographically representative of all coaches in the U.S., so the data may not accurately reflect the experiences of all coaches. Importantly, selection bias is present, given that perhaps more effective coaches (or ones who may feel more impactful) responded to the survey. These coaches also may represent organizations who value PYD approaches and/or recruit coaches who are more effective. Additionally, White, male coaches were overrepresented in this sample. Future studies should seek more diverse participants to enhance generalizability and elevate the perspectives of underrepresented coaches. Further, data were cross-sectional and self-reported, so causation cannot be established between the predictor variables and coaches’ perceived impact.

Common method bias is a potential limitation because the independent and dependent variables were measured using a single self-report survey with similar response formats (64). As a result, associations among variables may have been inflated by shared measurement methods, and social desirability may have influenced coaches’ self-reported perceptions. However, several aspects of the study design may have reduced this risk. Many of the predictor variables were designed as objective (e.g., coaching certification, paid coaching status, performance evaluation, and coaching setting), making them less susceptible to response style bias than purely perceptual measures (64). In addition, participant anonymity was emphasized throughout data collection, and the dependent variable was positioned later in the survey following demographic and contextual questions, reducing the likelihood of immediate consistency effects.

Although there is increasing evidence supporting the use of single-item measures when constructs demonstrate adequate conceptual clarity (52), several variables in this study were measured using a single item. This approach may have limited construct validity and increased measurement error for some variables. While survey items were informed by prior research and refined before inclusion in the U.S. National Coach Survey, future research should further develop and validate multidimensional measures, particularly for coaches’ perceived impact on athlete life skill development. Finally, the outcome examined in this study reflects coaches’ perceptions of their influence rather than athletes’ actual life skill development. As such, conclusions should be interpreted cautiously. Other important PYD outcomes, including caring relationships, sense of belonging, and social support, were not assessed. Future research should incorporate multiple informants (e.g., athletes, parents, and observers), longitudinal designs, and direct observations of coaching practices to better understand coaches’ contributions to life skill development and transfer over time. In addition, the predictors examined in this study were not exhaustive. Future research should consider additional coach, athlete, organizational, and sport-context characteristics that may explain greater variance in coaches’ perceived impact.

## Conclusion

The present study offers several insights into factors that may shape coaches’ impact on athlete life skill development. By analyzing a unique national dataset, this study distills important relationships among contextual, community, and organizational factors and self-reported coach perceived impact on athletes’ life skills. Importantly, organizational factors were strongly related to coaches’ perceived impact on athletes’ life skills. As such, athletic departments or other sport organizations may seek training, require certifications, provide regular evaluations, and offer financial compensation to coaches to create a more supportive environment for coaches, potentially contributing to broader athlete outcomes inclusive of life skill development.

## Data Availability

The raw data supporting the conclusions of this article may be made available by the authors upon reasonable request, pending an appropriate data use agreement and in accordance with data-sharing practices approved by the institution's Institutional Review Board (IRB).
